# *Bombus terrestris terrestris* (Linnaeus, 1758) and hybrids with the endemic *Bombus xanthopus* spotted on Capraia Island (Tuscan Archipelago, Italy): some conservation management implications

**DOI:** 10.1007/s00114-023-01843-y

**Published:** 2023-04-12

**Authors:** Chiara Benedetta Boni, Francesca Coppola, Marino Quaranta, Francesca Giannini, Antonio Felicioli

**Affiliations:** 1 Department of Veterinary Sciences, University of Pisa, Viale delle Piagge 2, 56124 Pisa, Italy; 2 Interdepartmental Center of Agro-Environmental Research “Enrico Avanzi”, University of Pisa, Via Vecchia di Marina 6, 56122 Pisa, Italy; 3 CREA Research Centre for Agriculture and Environment, Via di Saliceto 80, 40128 Bologna, Italy; 4 Tuscan Archipelago National Park, Loc. Enfola, 57037 Portoferraio, LI Italy

**Keywords:** Bumblebees, Recolonization, Bumblebees mass-migration, Distribution range, Hybrid, Conservation

## Abstract

Expansion of wild and managed allochthonous species leads to potential negative consequences for the endemic wildlife, such as resource competition, pathogens spread, hybridization and native species replacements. On Capraia Island, the last sighting of *Bombus terrestris terrestris* dates back to 1917. All subsequent surveys carried out on the island only reported the presence of *B. xanthopus* and *B. pascuorum melleofacies* with *B. t. terrestris* apparently no longer existing in the area. In 2021 *B. t. terrestris* was again detected on the island raising two main hypotheses: (i) *B. t. terrestris* has always been present with a low population density, such as not to be detected in previous investigations, or (ii) its presence is the result of a more recent recolonization. The recolonization event may be promoted by either intentional or unintentional introduction or it may be the result of a natural migration from the mainland or surrounding islands. Hybridization between *B. t. terrestris* × *B. xanthopus* on Capraia Island has been also ascertained by the detection of hybrid queens, workers and males. These new finding provides insight on the distribution range of *B. t. terrestris* in the Tuscan Archipelago and raise concern on the conservation of the endemic *B. xanthopus* population.

## Introduction

*Bombus terrestris* (L. 1758) is a ubiquitous Palearctic species, occurring throughout Europe with a very high population density (Intoppa et al. 1995; Rasmont et al. 2008). In Europe seven morphological subspecies of *B. terrestris* are present: *B. t. terrestris* in continental Europe; *B. t. audax* Harris, 1780, in British Islands; *B. t. calabricus* Krüger, 1956, in South Italy and Sicily; *B. t. canariensis* Pérez, 1895, in Canary Islands; *B. t. dalmatinus* Dalla Torre, 1882, in farthest South-East France, North Italy, Balkanic Peninsulas and surrounding regions; *B. t. lusitanicus* Krüger, 1956, in South-West France, Iberian Peninsula, Balearic Islands and Madeira; *B. t. sassaricus* Tournier, 1890, in Sardinia (Rasmont et al. 2008). *B. xanthopus* Kriechbaumer, 1870, present in Corsica, Elba and Capraia Islands and considered as a subspecies of *B. terrestris* until 2015 (Rasmont et al., 2008), has been elevated to the status of endemic Corsican species due to its molecular and eco-chemical features (Lecocq et al. 2015; Rasmont et al., 2021). Nevertheless, the acceptance of the species assignment for the taxon *xanthopus* seems to be provisional, as the Poisson-tree-process (PTP) provides conflicting results, leaving the taxonomic nomenclature a still open issue (Williams, 2021). The phenology of *B. t. terrestris* is usually characterized by one queen generation and a flying period ranging from March to August (Pawlikowski et al. 2020). However, *B. t. terrestris* population in Mediterranean regions, characterized by mild climates, shows flexibility in phenology compared to the inland population. Such flexibility result in all year-round activity with two generations, one in autumn and the other in winter (Rasmont 1985; Ricciardelli d’Albore 1986; Rasmont and Adamski 1995; Rasmont et al. 2008), as also demonstrated in laboratory conditions (Beekman 1998; Beekman and Van Stratum 2000). Since the end of the 1980s, colonies of *B. t. terrestris*, *B. t. sassaricus* and *B. t. dalmatinus* have been reared and commercialized throughout Europe and Italy to increase the efficiency of pollination in greenhouse crops (i.e. tomatoes, strawberries, blueberries) (Velthuis and Van Doorn 2006; Chandler et al. 2019). Bumblebee commercialization has led to introduction of allochthonous subspecies with consequent alteration of the subspecies distribution range (Ings et al. 2005; Velthuis and Van Doorn 2006; Ghisbain et al., 2021). Colonies commercialization for crop pollination purpose and accidental importation in non-European countries also led to the establishment of feral colonies of *B. terrestris* in Tasmania (Semmens et al., 1993; Buttermore 1997), in Israel (Dafni and Shmida 1996) and in Chile (Ruz 2002), as well as in countries where it has now become naturalized, such as New Zealand (Goulson 2003) and Japan (Matsumura et al. 2004).

In Italy, four subspecies of *B. terrestris* exist: *B. t. terrestris* and *B. t. dalmatinus* Dalla Torre, 1882; *B. t. calabricus* Krüger, 1958; and *B. t. sassaricus* Tournier, 1890 (Rasmont et al. 2008). *B. terrestris* subspecies show differences in the colour pattern, behavioural traits (i.e. aggressiveness, colony dimensions, foraging performance) (Rasmont et al. 2008; Ings et al. 2010) and physiological features (De Jonghe 1986; Chittka et al. 2004). *B. t. terrestris* has a black coat with a yellow collar on the thorax, a yellow band on the abdomen (which has a white tip) and black legs (Ings et al. 2005). This colour pattern is very similar to that of other *Bombus* species present in Italy such as *B. cryptarum* (Fabricius, 1775) and *B. lucorum* (Linnaeus, 1761), the latter present only at altitudes over 500 m (Intoppa et al. 2009).

*B. t. terrestris* is widespread throughout mainland Tuscany at low altitudes and in the Archipelago (Intoppa et al. 1995; Rasmont and Quaranta 1997; Generani et al. 2001). The Tuscan Archipelago (Italy) includes seven islands, Gorgona, Capraia, Elba, Pianosa, Montecristo, Giglio and Giannutri extending along the north-south axis of Tyrrhenian Sea for a total of 295 km^2^ (www.islepark.it). Since the end of the 1970s, the presence of *B. t. terrestris* has been ascertained on all Tuscan Archipelago islands (Fanfani and Groppali 1979; Rasmont and Adamski 1995; Rasmont and Quaranta 1997; Generani et al. 1998, 2001; Cini et al. 2022) except Capraia. On Capraia Island, the last sighting of *B. t. terrestris* dates back to 1917 (Razzauti 1917). Since then, only *B. xanthopus* and *B. pascuorum melleofacies* were detected (Masi 1933; Rasmont and Adamski 1995; Rasmont and Quaranta 1997; Generani et al. 2001). Within a wider project focused to implement a monitoring network of bee species in the Tuscan Archipelago National Park and increase the knowledge on the ecology, biology and conservation status of these pollinators, the aim of this work was to assess the status of *B. t. terrestris* on Capraia Island and its potential impact on the endemic population of *B. xanthopus*.

## Materials and methods

**Fig. 1:**
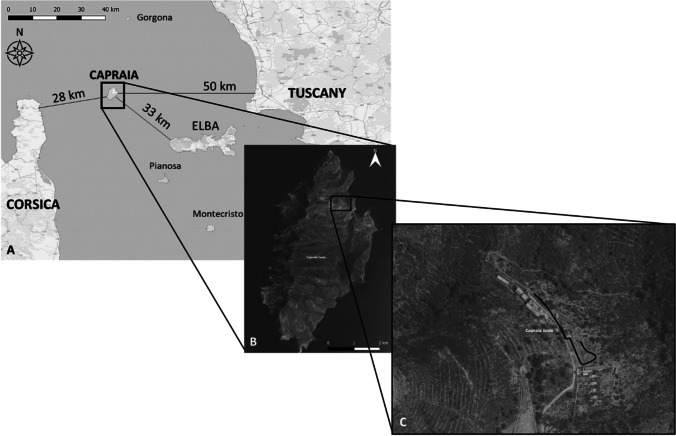
**A** Map of the Tuscan Archipelago, black lines indicate distances between Capraia and Elba, Corsica and Tuscany coast, **B** enlarged view of Capraia Island, the black square indicates the area in which bee fauna survey was performed and **C** detail of the survey area, the black line indicates the 250 m long transect on which bees were collected.

In summer-autumn 2021 and winter-spring 2022, a survey of bee (Apoidea: Anthophila) fauna in the Tuscan Archipelago National Park was performed (Ministero dell’Ambiente e della Tutela del Territorio e del Mare 2019). On Capraia Island (19 km^2^), a transect 250 m long and 2 m wide (45° 05′ 49.5″N; 9° 83′ 18.6″E) in Loc. Porto Vecchio (150 m a.s.l.) was defined according to guidelines for European pollinator monitoring scheme (Potts et al. 2020) (Fig. 1). The transect site was defined in agreement with the Tuscan Archipelago National Park to cover an area as representative as possible of the island’s environment (i.e. the presence of cultivated areas such as vineyards and Mediterranean shrub). The transect was walked once a month in May, July and September 2021 and in February and March 2022 for a total of 5 capture sessions performed, each lasting 1 h (i.e. from 12:00 to 13:00). During transect sampling, all present bees were captured by using an entomological net including specimens phenotypically attributable to *B. t. terrestris*. Each captured individual was placed in a 50-ml falcon tube containing chopped cork, euthanized by adding two drops of ethyl acetate and stored at −20°C until preparation for taxonomic identification. To each sample, an individual code containing capture information (i.e. date, site, and identification number of capture) was attributed. Taxonomic recognition was performed only on taped bumblebees individuals based on analysis of diagnostic morphological traits (Rasmont et al. 1986), hair and cuticle colours (Ruz 2002; Rasmont et al. 2008) using the identification key of Rasmont et al. (2021).

## Results

**Fig. 2:**
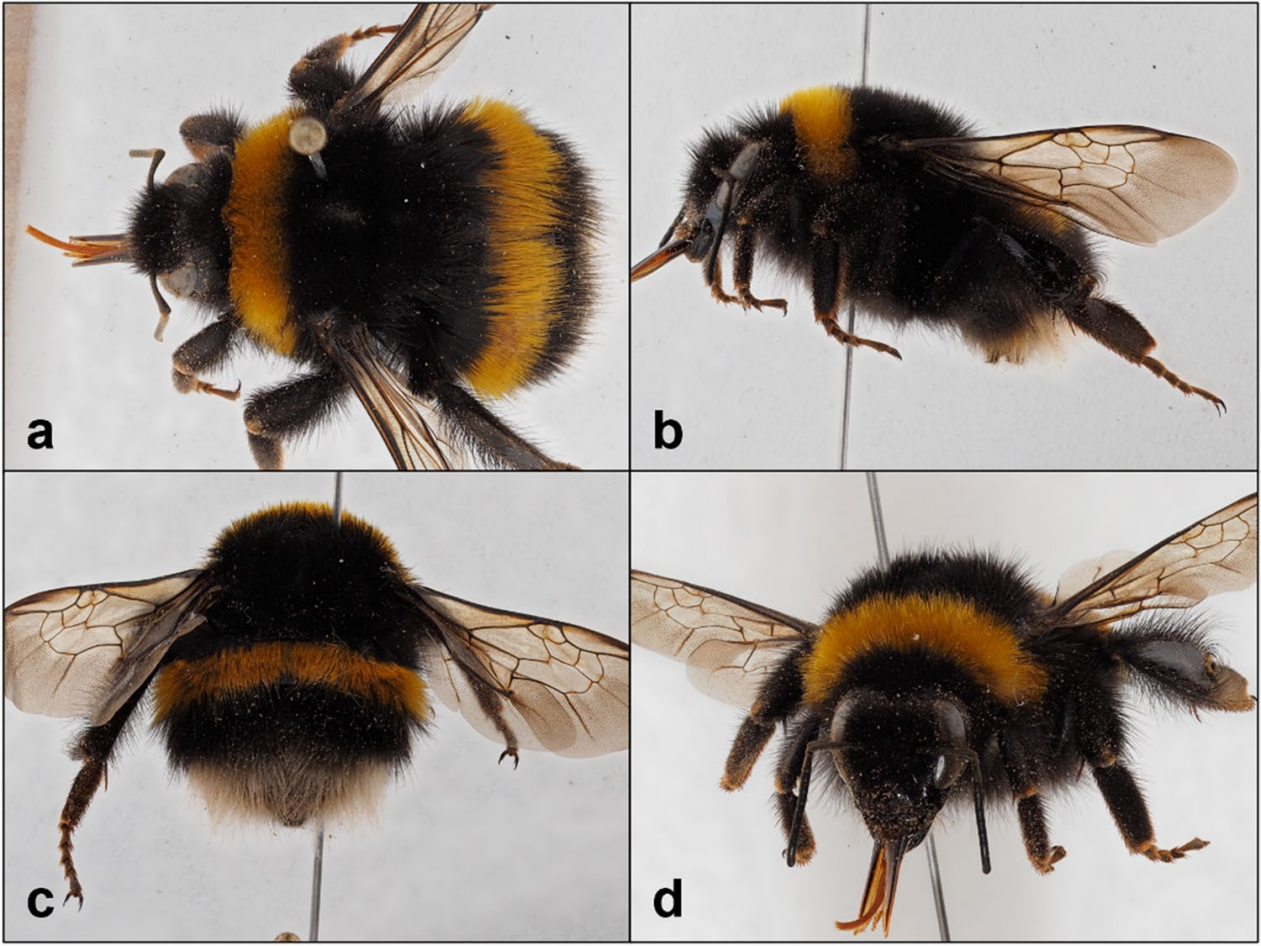
Queen of *Bombus terrestris terrestris* captured on Capraia Island in 2021. **a** Top view: broad yellow collar and yellow tergite 2 are visible; **b** side view: black legs cuticula and black corbicula bristles are visible; **c** rear view; **d** frontal view of the specimen.

**Fig. 3:**
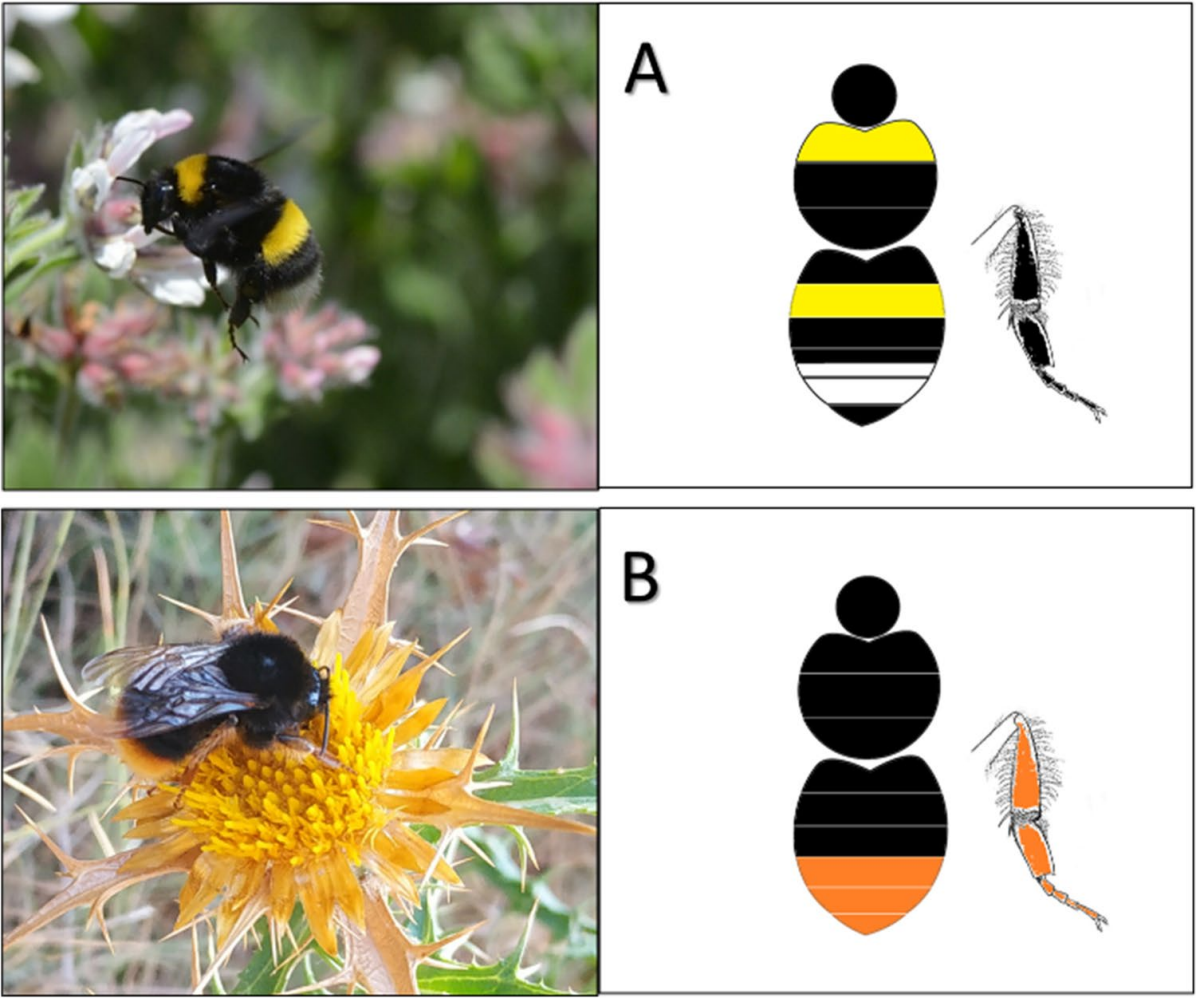
**A** Queen of *Bombus terrestris terrestris* and its schematic colour pattern of *B. terrestris terrestris*: yellow collar and tergite 1, black legs cuticula and black corbicula bristles. **B** Worker of *B. xanthopus*, and its schematic colour pattern of *B. xanthopus*: black thorax, few yellow hairs on tergite 1, reddish legs cuticula and corbicula bristles.

During the survey 161 bees were collected of which 65 (40.4%) bumblebees, 20 in 2021 and 45 in 2022 (Table 1). *B. t. terrestris* was detected on Capraia in both 2021 and 2022: 1 ♀ (queen), 21.IX.2021, C.B. Boni leg., M. Quaranta det.; 2 ♂ 15.II.2022, F. Coppola leg., M. Quaranta det.; and 6 ♂ 1♀ (worker), 22.III.2022, F. Coppola, M. Quaranta det. for a total of 10 (15.4%) captured individuals. The collected specimens presented the following colour pattern: black facial and vertex hair; broad yellow collar, extending slightly below the tegulae, with isolated and sparse black hair at the margins of the collar; the rest of the thorax covered with black hair; legs with black cuticle covered with short black hair; black corbicula bristles; tergite 1 and tergite 3 black; tergite 2 yellow; tergite 4 and tergite 5 ivory white; and tergite 6 covered with short black hair (Fig. 2 and Fig. 3A). The 36.9% (*n*=24) of collected bumblebees during survey were assigned to *B. xanthopus*: 4 ♀ 1 ♀ (queen), 16.V.2021, F. Coppola leg., M. Quaranta det.; 3 ♀ 1 ♂, 18.VII.2021, C.B. Boni leg., M. Quaranta det.; 6 ♀ 1 ♂, 15.II.2022, C.B. Boni leg., M. Quaranta det.; and 8 ♀, 22.III.2022, F. Coppola leg., M. Quaranta det.. All the *B. xanthopus* specimens present black thorax with collar missing or restricted to a few yellow hairs; tergite 1 to tergite 3 entirely black or with restricted to a few yellow hairs; and tergite 4 to tergite 6 reddish and reddish cuticula with entirely reddish bristles (Fig. 3B).

**Table 1:** Number of queens (Q), workers (W) and males (M) of B. t. terrestris, B. xanthopus and B. xanthopus × B. t. terrestris hybrids collected on Capraia Island in 2021 and 2022

	2021				2022			
	Q	W	M	Total	Q	W	M	Total
*B. t. terrestris*	1	0	0	1	0	1	8	9
*B. xanthopus*	1	7	1	9	0	14	1	15
*B. xanthopus* × *B. t. terrestris*	1	6	3	10	1	11	9	21

**Fig. 4:**
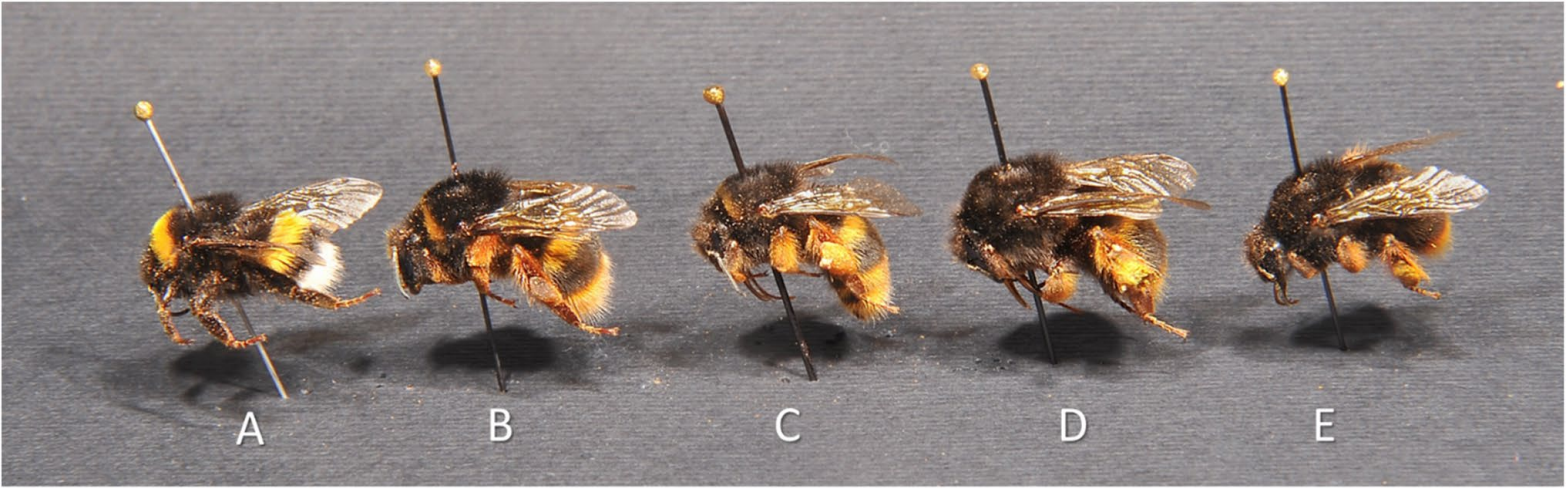
Worker of *B. t. terrestris*, *B. xanthopus* and of *B.t. xanthopus × B. t. terrestris* showing different intermediate colour pattern collected on Capraia Island. **A** *B. t. terrestris*: yellow collar and tergite 1, black legs cuticula and black corbicula bristles; **B** *B. xanthopus × B. t. terrestris*: yellow collar and tergite 1, reddish legs cuticula and corbicula bristles; **C** *B. xanthopus × B. t. terrestris* with few yellow hairs on collar, yellow tergite 1, reddish legs cuticula and corbicula bristles; **D** *B. xanthopus × B. t. terrestris:* absence of collar, yellow tergite 1, reddish legs cuticula and corbicula bristles; **E** *B. xanthopus*: black thorax, few yellow hairs on tergite 1, reddish legs cuticula and corbicula bristles.

On the Island, also hybrid specimens between *B. xanthopus* × *B. t. terrestris* were found in both years of survey for a total of 31 (47.7%) specimens: 6 ♀ 1 ♀ (queen) 1 ♂, 16.V.2021, F. Coppola leg., M. Quaranta det.; 2 ♂, 18.VII.2021, C.B. Boni leg., M. Quaranta det.; 5 ♀ 4 ♂, 15.II.2022, C.B. Boni leg., M. Quaranta det.; and 6 ♀ 1 ♀ (queen) 5 ♂ 22.III.2022, F. Coppola leg., M. Quaranta det.. Hybrids individuals presented different intermediate colour patterns between *B. xanthopus* and *B. t. terrestris* but always with reddish legs cuticula with entirely reddish cuticula bristles (Fig. 4).

## Discussion

Results obtained in this investigation provide new knowledge on *B. t. terrestris* distribution range within the Tuscan Archipelago and confirm the presence of this subspecies also on Capraia Island. Such finding occurs more than 100 years from Razzauti (1917) sighting. The detection of male, worker and queen individuals suggests the presence of at least one reproductive colony on the island. Two main hypotheses can be formulated for the presence of this bumblebee subspecies on Capraia: (i) *B. t. terrestris* has always been present on the Island with a low population density, such that it has gone unnoticed by other authors, or (ii) its presence is the result of a more recent colonization promoted by several factors such as intentional importation for pollination, unintentional transport by ferries and boats or by mass-migrating phenomena.

Although Razzauti (1917) does not provide any indication on the number of observed individuals or abundance, a low population density of *B. t. terrestris* could be hypothesized on Capraia since no other authors reported its presence in their checklist (Masi 1933; Rasmont and Adamski 1995; Rasmont and Quaranta 1997; Generani et al. 2001). Moreover, the absence of hybrids between *B. xanthopus* and *B. t. terrestris* on the island after Razzauti (1917) sighting of *B. t. terrestris*, allows to hypothesize that occurrence of this subspecies might have been an isolated and unique event.

Agriculture on Capraia Island is mainly based on olive growing, viticulture and fruit growing. Horticulture is only performed for family use and no greenhouses are present on the island (Felicioli 2021). Therefore, it is unlikely that *B. t. terrestris* has be imported on Capraia for pollination purposes. Conversely, the unintentional transport of *B. t. terrestris* individuals from Tuscan coasts or other Archipelago islands by ferries and boats that daily connect these areas cannot be excluded. Furthermore, a recent study performed in Northern Europe demonstrated that bumblebees are able to travel over long distances (up to 200 km), even crossing large water bodies via active flight aided by favourable wind currents (Fijen 2021). Individuals of the white-tailed species complex, such as *B. terrestris*, *B. lucorum* (Linnaeus, 1761) and *B. ruderatus* (Fabricius, 1775), were frequently observed flying at sea at about 50 km from the coast, either coming from the sea or flying towards the sea (Mikkola 1978; Fijen 2021). Capraia Island is 30 km from Elba, 40 km from Gorgona and 50 km from the coast of Tuscany, areas where *B. t. terrestris* is present. These distances are compatible with those travelled by bumblebees in Northern Europe (Fijen 2021). Therefore, migration from near islands or from the Tuscan coast is a solid hypothesis, and a phylogenetic analysis should be performed to better explain the origin of *B. t. terrestris* individuals present on Capraia Island. Little is known about the ecological and biological factors that determine bumblebee mass-migration. Data on migration timing and reasons on factors driving migration route and the choice of settle area are desirable. This bumblebee newly observed behavioural trait could strongly influence bumblebee distribution ranges and implies a renewal in the planning of conservation initiatives and in the management of endangered species. Further investigation on recolonization trends of bumblebee species or subspecies in areas of ancient presence is also need.

The distribution of *B. xanthopus* on Capraia Island is well known and documented since 1933 (Masi 1933), and its presence was also confirmed during this investigation. In this investigation, hybrids individuals between *B. t. terrestris* and *B. xanthopus* were recorded on Capraia Island for the first time, which indicates that warnings highlighted by Williams (2021) came true. Currently, no data on the abundance of *B. xanthopus* population on Capraia are available. However, considering the small dimension of the island, the occurrence of hybridization between *B. t. terrestris* and *B. xanthopus*, as already occurred on Elba Island (Kruger 1954; Rasmont and Adamski 1995; Rasmont and Quaranta 1997) and reported on the Tuscan coast (Quaranta and Felicioli 2012), is not surprisingly and raising concern on the conservation of the endemic *B. xanthopus* population. Impacts of the introduction of *B. terrestris* on native species have been already evaluated and documented worldwide (Inari et al. 2005; Winter et al. 2006; Dafni et al. 2010; Russo 2016). In Japan, imported *B. terrestris*, now considered naturalized, mate with a high frequency with the endemic *B. hypocrita* Pérez, 1905, producing inviable hybrid eggs and determining the potential decline of the native bumblebee (Kanbe et al. 2008; Kondo et al. 2009; Tsuchida et al. 2010, 2019). Investigation on interspecific hybridization between *B. terrestris* and *B. ignitus* shows that males of *B. ignitus* Smith, 1869, mate favourably with *B. terrestris* queens, leading to a potential genetic contamination of the endemic species (Yoon et al. 2009). In addition to genetic contamination, allochthonous *B. terrestris* could threaten the endemic population by competing for nest sites, spreading parasites and pathogens and inducing disturbances to the reproduction of the local flora (Hingston and McQuillan 1998; Matsumura et al. 2004; Yoneda et al. 2008; Nagamitsu et al. 2009; Dafni et al. 2010; Cilia et al. 2022).

Despite controversies over the taxonomy of *B. xanthopus*, the presence of hybrids individuals on the Island assumes relevance either they derive from an inter- or an intra-specific hybridisation. Fertility of hybrids *B. t. terrestris* × *B. xanthopus*, as well as *B. t. terrestris* × *B. t. canariensis*, has been yet demonstrated under laboratory condition (De Jonghe 1986; van den Eijnde and de Ruijter 2000), and for this reason, a potential spread of *B. t. terrestris* in Corsica and Canary Islands could cause threat to the endemic *B. xanthopus* and B*. t. canariensis*, respectively (Williams, 2021; Ghisbain et al., 2021). In this case, the risk of genetic contamination of the native subspecies could be potentially higher considering that mass-migration of *B. t. terrestris* in Mediterranean area could happen twice a year. Despite the general decline of pollinators worldwide, some species are recording expansion of their distribution areas, especially due to climate change and commercialisation for pollination purpose (Ghisbain et al., 2021), and in this context, *B. t. terrestris* could pose a severe threat for the genetic conservation of the endemic *B. xanthopus*.

Moreover, the presence of hybrids may increase the probability of the loss of the endemism throughout genetic contamination or the potential sterility of hybrid males.

The detection on Capraia Island of hybrids males indicates that hybrid queens in field condition can at least develop and lay haploid eggs. This evidence arises the urgency to establish the species status of the taxon *xanthopus* and then the fertility status of hybrids males since the presence of sterile males could act in a similar way of the male-sterile release technique.

In conclusion, results obtained in this investigation clearly indicate the presence of *B. t. terrestris* and *B. t. terrestris* × *B. xanthopus* hybrids on Capraia Island. These evidences raise several concerns about conserving endemic *B. xanthopus* populations that should prompt assessing the spread, the genetic origin and/or colonization pathways of *B. t. terrestris* on the island as well as the fertility status of hybrids. Furthermore, genetic analyses of hybrid individuals to establish the degree of hybridization and to exclude that intermediate phenotypes found on the Island may actually be part of the natural intraspecific variations of *B. xanthopus* population, are necessary. Resulting data will provide a useful tool for the development of a conservative action plan.

## Data Availability

All data are available from the corresponding author
